# Targeted Morphoproteomic Profiling of Ewing's Sarcoma Treated with Insulin-Like Growth Factor 1 Receptor (IGF1R) Inhibitors: Response/Resistance Signatures

**DOI:** 10.1371/journal.pone.0018424

**Published:** 2011-04-06

**Authors:** Vivek Subbiah, Aung Naing, Robert E. Brown, Helen Chen, Laurence Doyle, Patricia LoRusso, Robert Benjamin, Pete Anderson, Razelle Kurzrock

**Affiliations:** 1 Department of Pediatrics, The University of Texas MD Anderson Cancer Center, Houston, Texas; 2 Department of Investigational Cancer Therapeutics (Phase I Clinical Trials Program), The University of Texas MD Anderson Cancer Center, Houston, Texas; 3 Department of Pathology and Laboratory Medicine, UT Health, University of Texas–Houston Medical School, Houston, Texas; 4 Cancer Therapy Evaluation Program, National Cancer Institute, Bethesda, Maryland; 5 Wayne State University–Barbara Ann Karmanos Cancer Institute, Detroit, Michigan; 6 Division of Cancer Medicine, Department of Sarcoma Medical Oncology, University of Texas MD Anderson Cancer Center, Houston, Texas; Dana-Farber Cancer Institute, United States of America

## Abstract

**Background:**

Insulin-like growth factor 1 receptor (IGF1R) targeted therapies have resulted in responses in a small number of patients with advanced metastatic Ewing's sarcoma. We performed morphoproteomic profiling to better understand response/resistance mechanisms of Ewing's sarcoma to IGF1R inhibitor-based therapy.

**Methodology/Principal Findings:**

This pilot study assessed two patients with advanced Ewing's sarcoma treated with IGF1R antibody alone followed by combined IGF1R inhibitor plus mammalian target of rapamycin (mTOR) inhibitor treatment once resistance to single-agent IGF1R inhibitor developed. Immunohistochemical probes were applied to detect p-mTOR (Ser2448), p-Akt (Ser473), p-ERK1/2 (Thr202/Tyr204), nestin, and p-STAT3 (Tyr 705) in the original and recurrent tumor. The initial remarkable radiographic responses to IGF1R-antibody therapy was followed by resistance and then response to combined IGF1R plus mTOR inhibitor therapy in both patients, and then resistance to the combination regimen in one patient. In patient 1, upregulation of p-Akt and p-mTOR in the tumor that relapsed after initial response to IGF1R antibody might explain the resistance that developed, and the subsequent response to combined IGF1R plus mTOR inhibitor therapy. In patient 2, upregulation of mTOR was seen in the primary tumor, perhaps explaining the initial response to the IGF1R and mTOR inhibitor combination, while the resistant tumor that emerged showed activation of the ERK pathway as well.

**Conclusion/Significance:**

Morphoproteomic analysis revealed that the mTOR pathway was activated in these two patients with advanced Ewing's sarcoma who showed response to combined IGF1R and mTOR inhibition, and the ERK pathway in the patient in whom resistance to this combination emerged. Our pilot results suggests that morphoproteomic assessment of signaling pathway activation in Ewing's sarcoma merits further investigation as a guide to understanding response and resistance signatures.

## Introduction

Ewing's sarcoma is the second most common malignant bone tumor in children, adolescents and young adults. Despite using a multimodal approach combining surgery, chemotherapy, and radiation, a therapeutic plateau has been attained with no change in overall survival [1], [2], [3], [4], [5]. Attempts to improve clinical outcome through collaborative trials beginning in the early 1970s sought to optimize care through ever more mechanistically-diverse chemotherapies. Strategies included increasing duration of treatment or dosage per cycle, decreasing treatment interval (i.e., interval dose compression), or using high-dose myeloablative chemotherapy followed by peripheral blood stem cell transplant [3]. However, survival remains poor for patients with metastatic disease. For metastatic Ewing's sarcoma at diagnosis, the risk of refractory or recurrent disease approaches 80% after initial therapy and the outcome of recurrent disease is poor with event-free survival less than 20% [3]. Treatment options for patients with refractory or recurrent Ewing's sarcoma are limited.

Early phase clinical trials frequently combine targeted agents to optimize benefit. Two challenges at the outset are 1) deciding which agents to combine given the heterogeneity of tumors and their various underlying resistance pathways and feedback loops, and 2) how to translate findings from the bench to the bedside or directly from the bedside [6]. Morphoproteomics (morphology+proteomics) involves immunohistochemical assessment of the activation of signaling pathways in cancer cells, and predicting susceptibility to small-molecule inhibitors, specific chemotherapeutic agents, and possibly, differentiating agents [7]. In some cases, drugs that fail early in the disease trajectory can produce renewed tumor regression later, particularly with rational addition of another drug [8]. Morphoproteomics can potentially identify targeted combinations of drugs appropriate for prospective testing [9].

Insulin-like growth factor 1 receptor (IGF1R)-targeted therapies have shown early promise [10], with responses in a small number of patients with Ewing's sarcoma [4], [11], [12], [13]. Currently available IGF1R antibodies recognize different epitopes of the receptor and, therefore, may exert different biological/clinical responses [14], [15]. Even so, phase I studies with different IGF1R antibodies demonstrated remarkable responses in a subset of Ewing's sarcoma patients [11], [12], [13]. While response rates in Phase II studies have not yet been reported, it is clear that while some responses have been dramatic, they occurred in only a minority of patients. The mechanisms underlying primary and secondary response and resistance are unknown.

Herein, we report our experience with two index cases of Ewing's sarcoma, with an initial positive response to an IGF1R inhibitor followed by resistance. Both patients subsequently responded to a combination of an IGF1R inhibitor and a mammalian target of rapamycin (mTOR) inhibitor. We performed morphoproteomic profiling to elucidate the functional signaling pathways in both patients.

## Methods

### Patient Selection, Treatment and Clinical Assessments

We reviewed the medical records of two patients with Ewing's sarcoma who were seen in the Phase I Clinical Trials Program at The University of Texas MD Anderson Cancer Center and initially treated with an IGF1R inhibitor alone, then subsequently with an IGF1R and mTOR inhibitor combination. The patients in this manuscript have given written informed consent (as outlined in the PLoS consent form) to publication of their clinical details. Treatment and consent on investigational trials, and data collection and morphoproteomic analysis were performed in accordance with the guidelines of the University of Texas MD Anderson Cancer Center Institutional Review Board (IRB).

The patients in the manuscript were derived from two different Phase I studies and a Phase II study using different IGF1R inhibitors and all the studies have been registered in www.clinicaltrials.gov. The scope of the studies, current status and clinical trial registration identifiers are as follows:

1. A Multiple Ascending Dose Study of R1507 in Patients with Advanced Solid Tumors (now closed and not actively recruiting patients) NCT00400361 (Phase I),

2. A Study to Determine the Activity of SCH 717454 in Subjects with Relapsed Osteosarcoma or Ewing's Sarcoma (Study P04720AM3) (now closed and not actively recruiting patients) NCT00617890 (Phase II),

3. IMC-A12 in Combination with Temsirolimus (CCI-779) in Patients With Advanced Cancers (Study is closed to Ewing's Sarcoma cohort) NCT00678769 (Phase I).

After initiation of an investigational therapy, patients were evaluated clinically at 3- to 4-week intervals. At each visit, a history was taken and physical examination performed along with comprehensive metabolic and hematologic panels. Patients were assessed for the onset of new symptoms and compliance with the investigational therapy. Tumor response was determined using Response Evaluation Criteria in Solid Tumors (RECIST) version 3.1 by positron emission tomography/computed tomography (PET/CT) scans or CT scans obtained about every six to eight weeks. Sections of original and recurrent tumor were available for analysis. The morphoproteomic analysis reported in this manuscript was not a part of the original Phase 1 trial protocols, and were carried out as a separate subsequent analysis. Patient consent and MD Anderson IRB approval were obtained for morphoproteomic analysis as outlined above.

### Immunohistochemical and Morphoproteomic Analysis

Immunohistochemical (IHC) probes were used to detect the following phosphorylated (p) antigens as published previously[16]: p-mTOR (Ser 2448); p-Akt (Ser 473);and p-extracellular signal-regulated kinase (ERK) 1/2 (Thr 202/Tyr204) [Cell Signaling Technology, Beverly, MA]; and p-signal transducer and activator of transcription (STAT)3 (Tyr 705) [Santa Cruz Biotechnology, Santa Cruz, CA]. In addition, IHC probes in specimens from the two patients were applied to detect the expressions of CD99 (DakoCytomation, Carpinteria, CA) and nestin (abcam, Cambridge, MA). Chromogenic signals were evaluated by brightfield microscopy and semi-quantified with regard to percentage of cells stained (0–100%) and the staining intensity (0: non-staining, 1+: weak staining, 2 +: moderate staining, and 3 +: strong staining). Subcellular compartmentalizations were assessed as plasmalemmal, cytoplasmic, and/or nuclear. Concurrently run positive and negative IHC controls reacted appropriately. The methods have been published previously [7], [9], [16], [17] and were performed in a laboratory that is certified under the Clinical Laboratory Improvement Amendments of 1988 (“CLIA”) as qualified to perform high-complexity clinical testing.

## Results

Patient outcomes with chemotherapy and targeted therapy are summarized below.

### 

#### Patient 1

A twenty-four year old Caucasian woman presented with a three-year history of back pain and left lower extremity pain. Magnetic resonance imaging (MRI) revealed a sacral mass, which was determined to be Ewing's sarcoma following pathological assessment at MD Anderson. The tumor cells were positive for CD99 and negative for chromogranin A, keratin and desmin. She underwent six cycles of intravenous chemotherapy through central line with vincristine (2 mg intravenous [IV]on day 1), adriamycin (37.5 mg/m2/day IV for 2 days), and ifosfamide (2500 mg/m2 IV for 4 days) with MESNA uroprotection, followed by resection of the tumor, confirmed as being Ewing's sarcoma. Fluorescent in situ hybridrization showed a positive result for a clone with an *EWSR1* gene rearrangement. The patient received postoperative radiation therapy, followed by six cycles of adjuvant chemotherapy with irinotecan. After six months of follow-up, lung metastases were discovered. She was started on etoposide and after five months, her tumors progressed. Liposomal doxorubicin (Doxil) was initiated, but stopped after tumor progression. The patient then underwent thoracotomy for removal of tumor, followed by erlotinib, followed by another lung resection.

**Figure 1:**
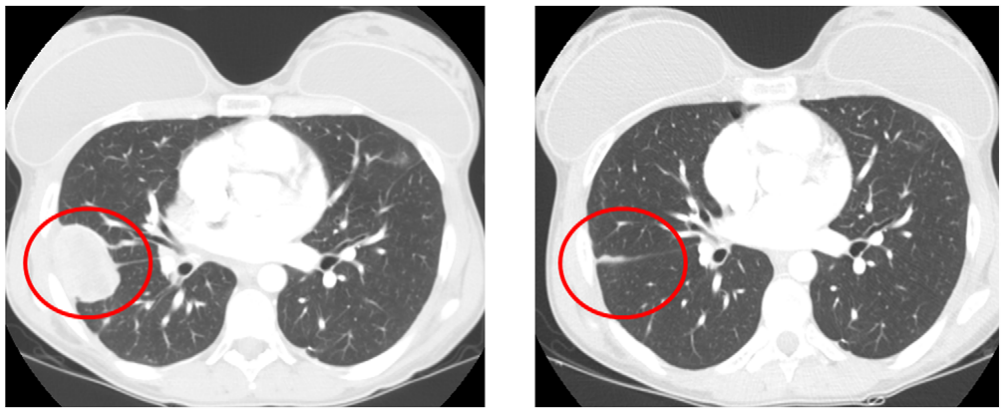
Imaging Responses in Ewing's sarcoma patient 1. CT of the thorax in patient 1 with Ewing's sarcoma response to IGF1R antibody (R1507) alone [12]. Left panel shows pre-treatment CT scan of the thorax showing metastatic Ewing's sarcoma in the lung. Right panel: Six weeks after IGF1R antibody (R1507) therapy shows regression of tumor.

**Figure 2:**
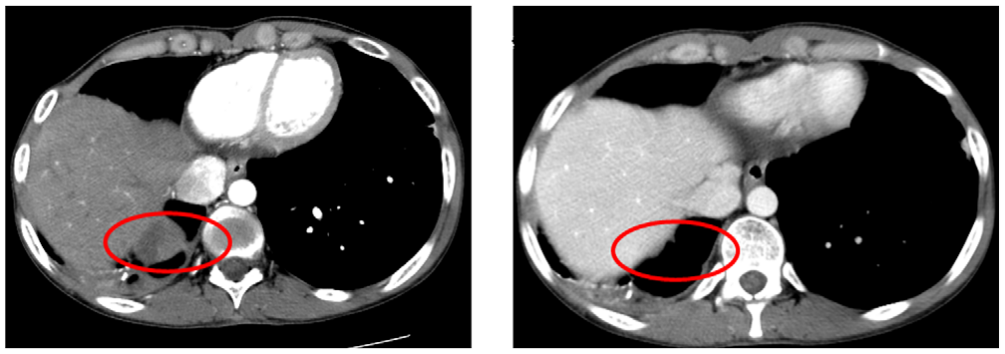
Imaging Responses in Ewing's sarcoma patient 1. CT of the thorax in patient 1 with Ewing's sarcoma response to IGF1R antibody (IMCA12)+ mTOR (Temsirolimus) combination. Left panel shows pre-treatment CT scan of the thorax showing metastatic Ewing's sarcoma in the lung. Right panel: Nine months after IGF1R antibody+ mTOR inhibitor (IMCA12+ Temsirolimus) therapy showing continued response.

She was then referred to the Phase I clinic at MD Anderson Cancer Center. A CT scan showed enlargement of numerous pulmonary metastatic lesions, the largest measuring 5.9 cm×5.1 cm. She was treated on three sequential Phase I trials, with continued disease progression. In December 2006, the patient was started on a Phase I study of R1507 (Roche, Nutley NJ), a fully human IgG1 type monoclonal antibody against IGF1R. Within six weeks, she had a dramatic response, with near complete tumor regression (Figure 1, previously described in [12]). No toxicity was noted. After 20 months of continued treatment a small focus of growing residual disease was found followed by surgical resection. Therapy continued for another 15 months, followed by progressed disease in the patient's lungs. She was started on another study using a different anti-IGF1R antibody (IMC-A12; Imclone, San Diego CA) [18] in combination with the mTOR inhibitor temsirolimus (NCT00678769). She tolerated this combination without any major side effects except a decrease in platelet counts. After 14 months of treatment, both PET/CT and chest CT scans show no disease (complete response) (Figure 2) and she continues on treatment.

#### Patient 2

**Figure 3:**
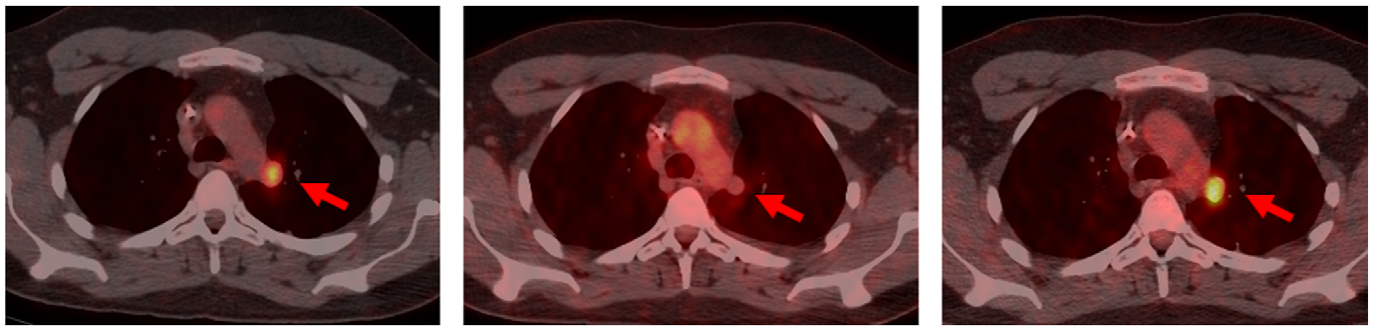
Imaging Responses in Ewing's sarcoma patient 2. FDG PET /CT in patient 2 with Ewing's sarcoma response to IMCA12+Temsirolimus combination and then emergence of resistance. Left panel shows pre-treatment FDG PET/CT scan of the thorax showing metastatic Ewing's sarcoma in the lung. Middle panel shows FDG PET/CT response after 8 weeks of treatment. Right panel shows re-emergence of resistance 16 weeks after IGF1R antibody+ mTOR (IMCA12+ Temsirolimus) therapy.

A twenty-one year old Caucasian man presented with back pain radiating to the left leg in December 2006. An MRI revealed a large left iliac mass extending into the sacroiliac joint. Biopsy showed Ewing's sarcoma with a t(11;22) translocation. On presentation he had a large left iliac primary lesion and bilateral pulmonary nodules and no evidence of bone marrow disease. He was enrolled on the Children's Oncology Group Study AEWS0031 (NCT00006734) and received standard q 3 week regimen consisting of vincristine (2 mg/m2 IV push, on day 1. maximum dose 2 mg.), doxorubicin(75 mg/m2/course continuous IV infusion over 48 hours, beginning day 1), cyclophosphamide (1200 mg/m2 IV infusion over 1 hour with MESNA uroprotection, on day 1), alternating with ifosfamide (1800 mg/m2/day IV infusion over 1 hour, Days 1–5 of each cycle. (9,000 mg/m2 max total dose) and etoposide (100 mg/m2/day IV infusion over 1 to 2 hours, days 1–5 of each cycle (500 mg/m2 total dose). His pain improved after one treatment and he had an excellent response. He then received 55.8 Gy radiation in 31 fractions to the pelvis for local control of the unresectable disease, as well as whole lung radiation therapy for his pulmonary nodules at the end of chemotherapy. He completed therapy with no evidence of disease. Eight months later he developed recurrent pulmonary nodules. A PET/CT scan showed activity only in lungs. He received topotecan and cyclophosphamide, and although his tumors responded initially, they eventually progressed. The patient then received temozolomide and irinotecan, without response, followed by enrollment on an IGF1R inhibitor study using SCH 717454 (Schering, Kenilworth NJ), an IGF1R antibody (19D12) [4], [19]. He had near complete response (18 of 19 nodules improving or disappeared) following 7 cycles. However, after 4 months, a solitary left lung nodule began to grow, and he was taken off study for progressive disease by RECIST. A thoracoscopic biopsy was done and confirmed Ewing's sarcoma. Subsequently, he was started on etoposide, but disease continued to progress. He then presented to the MD Anderson Phase I clinic and was enrolled on the protocol of IMC-A12, IGF1R antibody in combination with Temsirolimus, mTOR inhibitor (NCT00678769). Three out of four nodules showed a near complete response and one nodule remained stable. However, after four months, one nodule began to grow, and he was removed from study (Figure 3). The non-responding tumor was biopsied, and Ewing's sarcoma was confirmed. Subsequently, the patient was treated with high-dose ifosfamide and also received proton radiation therapy to the lung nodule.

### Morphoproteomics /Correlative Studies

The pre-treatment similarities and differences between patient 1 and 2 are shown in Table 1.

**Table 1:** Targeted Morphoproteomic Profiling of Ewing Sarcoma Patients Treated with Insulin-like Growth Factor-1 Receptor (IGF-1R) Inhibitor: Pretreatment Specimens.

	Ras/Raf kinase/Extracellular Signal-Regulated Kinase (ERK) Pathway*		mTORC2 Pathway*		STAT3 Pathway	Nestin
Patient No	CD99 (Plasmalemmal)	p-ERK ½ Thr 202/Tyr 204 [Nuclear]	p-Akt Ser 473 [Nuclear]	p-mTOR Ser2448[Nuclear];	p-STAT3** Tyr 705 [Nuclear]	(Cytoplasmic)
1	Present (1+)	Present (1–3+)	+/−	1+	<10%	0
2	Present (2+)	Present (0–3+)	2+	2+	>50%	1

*Maximum scoring intensity graded on a scale of 0 (no signal) to 3+ (high intensity).

**Percentage of positive tumoral nuclei.

#### Patient 1

**Figure 4:**
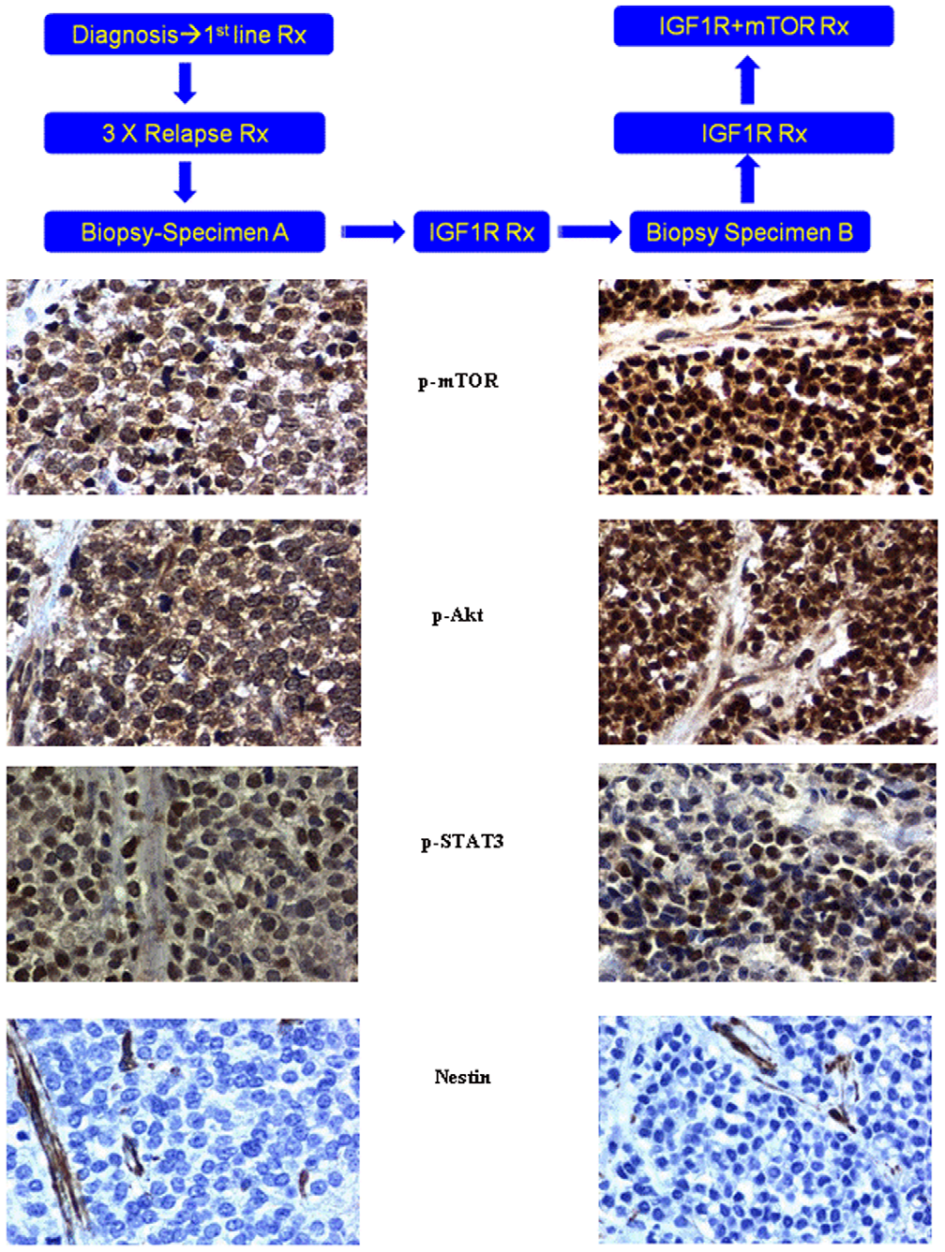
Schematic clinical history and immunohistochemistry of patient 1. Schematic history of patient 1 depicting time line of first biopsy(Biopsy A) and second biopsy (Biopsy B). Immunohistochemistry Patient 1. Pre-IGF1R treatment (Specimen A) and Post –IGF1R treatment (Specimen B) p-mTOR,p-AKT,p-STAT3 and nestin probes. Pre-treatment digital images (left hand frames, Specimen A) reveal: primarily cytoplasmic and plasmalemmal expression of p-mTOR (Ser 2448) and p-Akt (Ser 473) consistent with preponderance of mTORC1 pathway; occasional p-STAT3 (Tyr 705) in tumoral nuclei (up to ∼20%) and absence of cytoplasmic nestin. Post-treatment digital images (right hand frames, Specimen B) reveal: primarily nuclear p-mTOR (Ser 2448) and p-Akt (Ser 473) consistent with mTORC2 pathway preponderance; moderate increase in number of tumor cells with nuclear p-STAT3 (Tyr 705) (from 0 up to ∼50% in some regions) and absence of cytoplasmic nestin (endothelial cells serve as internal control). Original magnifications x400.

A limited number of sections of metastatic tumor from the following time points were available 1) before IGF1R antibody therapy; and 2) from the resistant recurrence that emerged during IGF1R antibody therapy (Figure 4). Constitutive mTOR pathway activation was noted in the pre-anti-IGF1R specimen (Figure 4) and reflected p-mTOR (Ser2448) expression in both cytoplasmic plasmalemmal and nuclear compartments. Similar findings have been reported in the Ewing's family of tumors [16]. There was also cytoplasmic plasmalemmal expression of p-Akt (Ser473), but with almost no nuclear expression, which was more consistent with P13K/Akt/mTORC1 signaling pathway activity. In contrast, there was apparent upregulation of these analytes in the specimen from the IGF1R-resistant tumor that emerged during IGF1R antibody therapy, with predominant nuclear expression of both p-mTOR (Ser2448) and p-Akt (Ser473), consistent with mTORC2 pathway activation [17], [20], [21]. In both biopsies, only endothelial cells were immunopositive for nestin, a neural precursor/differentiation marker, whereas the tumor cells were immunonegative. Notably, p-STAT3(Tyr705) was detected in a very minor component of the tumoral nuclei in the initial biopsy and was variably expressed in the post-anti- IGF1R treatment biopsy, ranging up to approximately one-half of the tumor cells in one microanatomical region.

#### Patient 2

**Figure 5:**
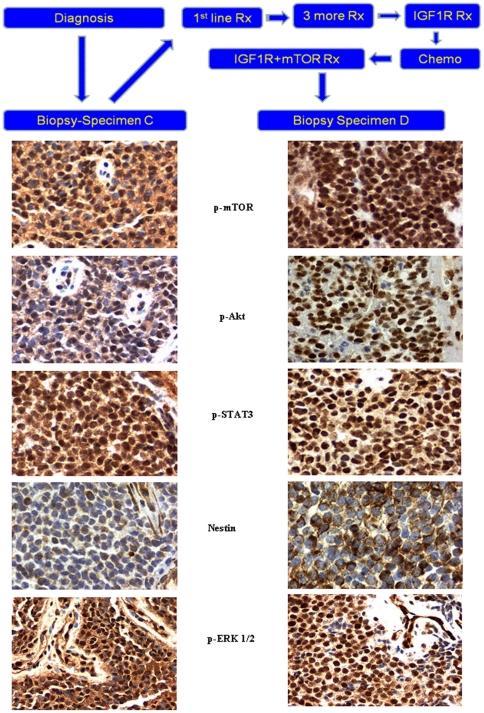
Schematic clinical history and immunohistochemistry of patient 2. Schematic history of patient 2 depicting time line of first biopsy (Biopsy C) and second biopsy (Biopsy D). Immunohistochemistry Patient 2. Pre-IGF1R treatment (Specimen C) and Post –IGF1R+ mTOR (Specimen D) p-mTOR,p-AKT,p-STAT3, nestin probes and p-ERK1/2. Pre-treatment digital images (left hand frames, Specimen C) reveal: primarily cytoplasmic but with some nuclear expression of both p-mTOR (Ser 2448) and p-Akt (Ser 473) indicative of both mTORC1 and mTORC2 pathways; nuclear p-STAT3 (Tyr 705) in the majority of tumoral nuclei and faint but detectable constitutive nestin expression. Post-treatment digital images (right hand frames, Specimen D) reveal: preponderance of nuclear p-mTOR (Ser 2448) and p-Akt (Ser 473) consistent with upregulation of the mTORC2 pathway; persistence of p-STAT3 (Tyr 705)expression in tumoral nuclei and an increase in cytoplasmic nestin expression, the latter consistent with temsirolimus therapy. Constitutive activation of the Ras/Raf /ERK pathway is noted in both specimens by p-ERK 1/2 (Thr202/Tyr204) expression, showing nuclear translocation. Original magnifications x400.

Sections of tumor before IGF1R antibody therapy and after IGF1R antibody combined with mTOR inhibitor therapy were available for analysis (Figure 5). A greater number of sections were available than for patient 1, permitting more extensive pathway evaluation (Figure 5).

**Figure 6:**
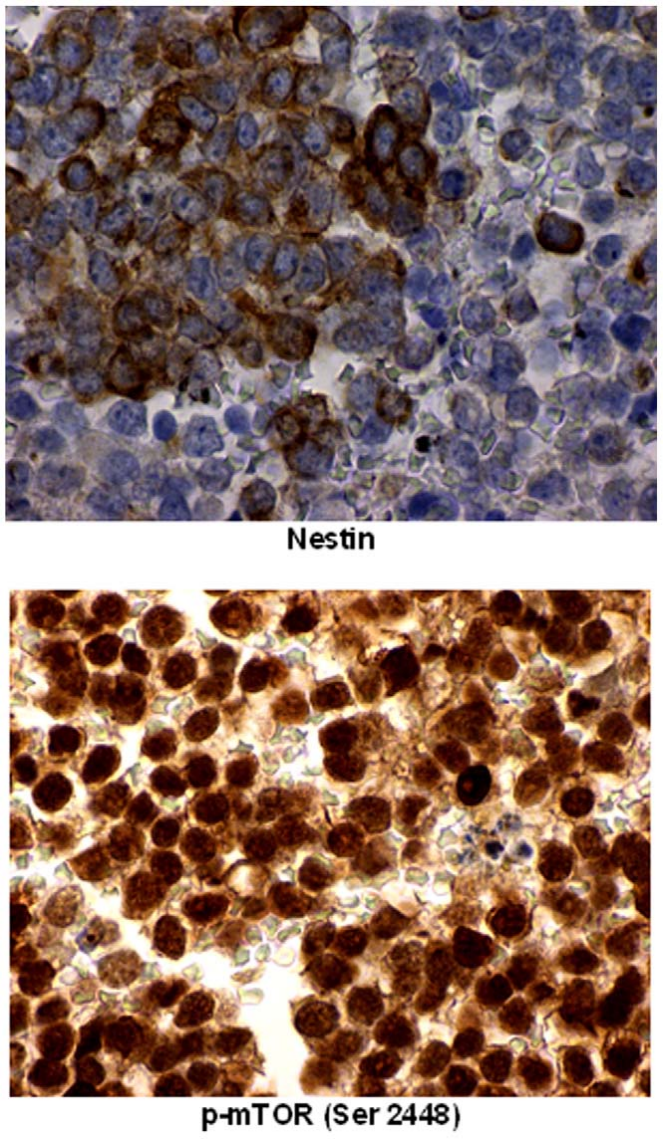
High magnification immunohistochemistry showing nuclear versus cytoplasmic staining. Post-IGF1R+ mTOR inhibitor therapy specimen from patient 2 in higher magnification (600X) showing brown chromogenic signal in nestin with predominantly cytoplasmic staining in the top panel and mTOR (Ser 2448) with predominant nulclear staining in the bottom panel.

There was constitutive activation of mTOR in the patient's original and recurrent tumor, evidenced by phosphorylation (p) of mTOR in a putative site of activation, Ser2448, with a predominantly nuclear distribution most likely indicating an mTORC2 complex (rictor + p-mTOR), [20] and correlative activation of Akt on Ser473 consistent with the presence of both mTORC2 and dominant expression of p-Akt (Ser473) in tumoral nuclei [17], [20], [21]. Constitutive activation of the Ras/Raf /ERK pathway is noted in both specimens by p-ERK 1/2 (Thr202/Tyr204) expression, showing nuclear translocation [9]. The expression appeared generalized and uniform in recurrent tumor and, to a lesser degree, in the original (primary) tumor. The signal transducer and activator of transcription (STAT)3 pathway was constitutively activated in both the primary and recurrent tumor, as evidenced by expression with nuclear translocation of p-STAT3 (Tyr 705) in the vast majority of tumor cells. The neural and endothelial precursor marker, nestin, was weakly expressed in the original (primary) tumor (0–1+). However, in the recurrent tumor, nestin was expressed in approximately 25% to 50% of tumor cells (up to 3+ cytoplasmic plasmalemmal expression) (Figures 5 and 6). Neural differentiation markers to CD56 and synaptophysin were expressed in recurrent tumor in the plasmalemmal and cytoplasmic compartments (not shown).

## Discussion

There is no effective therapy for advanced Ewing's sarcoma and patients with advanced metastatic disease succumb to their disease. Two patients with Ewing's sarcoma who responded, but then progressed after IGF1R inhibitor therapy alone showed consistently high mTORC2 expression in their tumors. Both patients responded after treatment to combined IGF1R and mTOR inhibition. The time interval between the first and the second IGF1R based therapy was 1 month for patient 1 and 4 months for patient 2. One patient had a continued response and has remained on IGF1R-based therapy for more than 50 months, the last 14 months of which has been an IGF1R inhibitor combined with an mTOR inhibitor. The patient's last imaging scans showed no disease. Unfortunately, the second patient acquired resistance.

Preclinical studies have shown that mTOR is a bypass pathway for IGF1R targeting. Similarly, combined inhibition of IGF1R and mTOR may circumvent counterproductive rapamycin-induced upregulation of Akt that can occur within 6 hours of treatment [3], [22]. Several Phase I/II clinical trials are currently investigating this potential synergy in advanced malignancies. (Clinicaltrials.gov identifiers: NCT00678769; NCT00880282 and NCT01016015).

**Figure 7:**
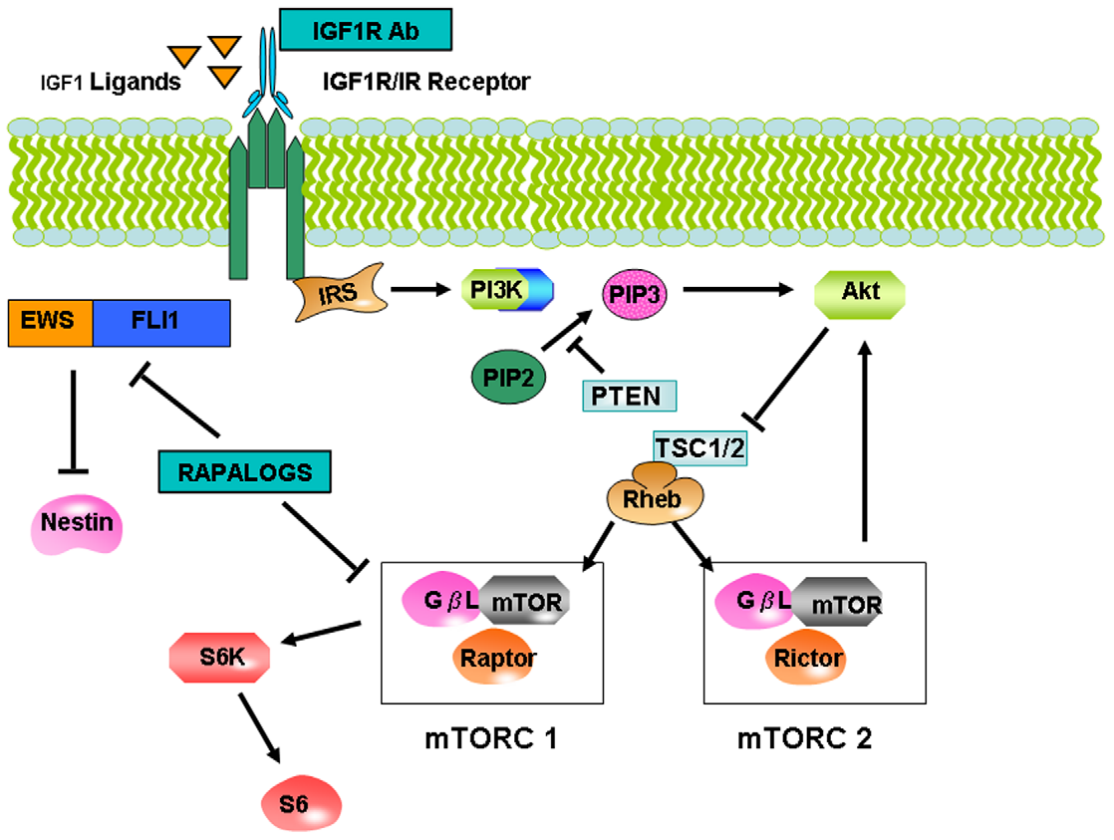
Simplified Insulin-like growth factor 1 receptor (IGF1R) and mTORC1 and mTORC2 signaling in relation with EWS-FLI1. Insulin Receptor substrate 1 (IRS1) and Phosphatidylinositol 3-kinases (PI3K) are activated by Insulin and/or IGF1 signaling at the insulin receptor level. PDK1 and Akt are recruited to the plasma membrane by products of PI3K, PIP2, PIP3 (phosphatidylinositol 3,4,5 trisphosphate and phosphatidylinositol 3,4 bisphosphate). After this event there is phosphorylation and activation of Akt by mTORC2 complex (mTOR + mLST8+ Rictor). This leads to a chain of activation of numerous targets by Akt. The TSC1/2 complex is also phosphorylated in this fashion. By the mechanism inactivation of TSC2's GAP activity for the small G protein Rheb is initiated. Now mTORC1 complex (mTOR + mLST8+Raptor) is activated by GTP-bound Rheb and phosphorylates proteins like S6K. This starts a negative feedback loop to modulate auto-activity, through S6K-mediated pathway decrease in the activation of PI3K. IGF1R antibody inhibits IGF1R signaling and rapalogs (Temsirolimus in this case) inhibit mTORC1 short term, and eventually inhibit mTORC 2 with chronic exposure, and also suppresses EWS-FLI1 which drives tumorigenesis in Ewing's sarcoma. EWS-FLI1 suppresses nestin.

In this context, our finding of upregulated p-Akt (Ser473) and p-mTOR (Ser2448) in patient 1's resistant tumor that emerged following IGF1R antibody therapy is consistent with a resistance mechanism that could be related to upregulation of TORC2. The patient was, however, treated successfully with termsirolimus, a TORC1 inhibitor. Although short-term inhibition of TORC1 drives TORC2 formation and results in Akt activation, long-term TORC1 inhibition abrogates Akt expression through activation of S6K by PKD1 and also blocks TORC2 assembly [23], [24]. Activated S6K can down-modulate Akt by acting against insulin receptor substrate 1 proteins and P13K [23]. Although these pathways have often been depicted as linear, clearly there is a complex interplay among signaling elements (Figure 7). EWS-FLI1 fusion protein, the hallmark of Ewing's sarcoma, downregulates insulin-like growth factor binding protein 3, IGFBP3, and upregulates IGF-1 expression resulting in enhanced IGF1R [25]. Therefore, treatment with an IGF1R inhibitor may counteract EWS-FLI1-mediated upregulation of the insulin receptor (IR) /IGF1R machinery. Temsirolimus is a “rapalog” and rapamycin has been shown to downregulate the EWS-FLI1 fusion protein [26] possibly also lessening IR/IGF1R signaling, and therefore providing an additional pathway by which this molecule might be operative in this patient.

In patient 2, constitutive activation of Akt and mTOR is similar to that in patient 1's recurrent tumor, and is seen in baseline pre-treatment tumor and in tumor that was resistant to IGF1R and mTOR combination treatment. These observations suggest the possibility that the TORC2 pathway has a role in primary and recurrent tumor [17] (Figures 5, 6, and 7). After IGFR treatment alone, response was followed by resistance. Similarly, an initial response was followed by re-emergence of resistance following treatment with the IGFR-mTOR inhibitor combination. The mechanism of response to the IGFR and mTOR combination may be similar to that in patient 1, that is, through Akt and mTOR suppression that occurs with chronic temsirolimus exposure [23], [24]. The biologic activity of the mTOR inhibitor temsirolimus is further confirmed by the upregulation of nestin seen in the patient's IGFR/mTOR resistant tumor, since temsirolimus down-modulates EWS-FLI1, which would be expected to upregulate nestin [26], [27] (Figure 7). However, other pathways are operative in this patient's tumor, including Ras/Raf /ERK and STAT3 (Figure 5).The relative overexpression of CD99 in this patient is consistent with activation of this pathway (not shown). In addition, p-Akt and S6K are not downregulated by combined IGF1R and mTOR inhibition, perhaps due to Ras/Raf/ERK activation. These signals might account for tumor resistance [26]. Of interest, in addition to nestin the patient's recurrent tumor showed a propensity toward differentiation along neural lines, as demonstrated by increased expression of other neural markers such as CD56 or neural cell adhesion molecule and synaptophysin.

We have demonstrated resistance/response mechanisms by morphoproteomics in two patients with advanced Ewing's sarcoma. This needs to be analyzed retrospectively and validated prospectively in a larger dataset to allow more robust conclusions. Next generaration whole exome sequencing of patients with Ewing's sarcoma responding to IGF1R based treatment and reverse phase protein array analysis in patients acquiring resistance is underway and will help to decipher unidentified mechanisms and perhaps unravel novel mutations and genetic aberrations in the response and resistance pathways.

Our observations suggest that rational combinations of targeted therapy, which modulate multiple relevant pathways, may be useful in overcoming resistance in patients with Ewing's sarcoma. Inhibition of IGF1R and/or IGF1R and mTOR has resulted in significant clinical activity in patients with Ewing's sarcoma. Study of an IGF1R inhibitor combined with mTOR inhibitor is currently underway. Our pilot results suggests that morphoproteomic assessment of signaling pathway activation in Ewing's sarcoma merits further investigation as a guide to understanding response and resistance signatures.
